# Development of magnitude processing in children with developmental dyscalculia: space, time, and number

**DOI:** 10.3389/fpsyg.2014.00675

**Published:** 2014-06-27

**Authors:** Kenny Skagerlund, Ulf Träff

**Affiliations:** Department of Behavioral Sciences and Learning, Linköping UniversityLinköping, Sweden

**Keywords:** developmental dyscalculia, number processing, approximate number system, ATOM, time estimation, development

## Abstract

Developmental dyscalculia (DD) is a learning disorder associated with impairments in a preverbal non-symbolic approximate number system (ANS) pertaining to areas in and around the intraparietal sulcus (IPS). The current study sought to enhance our understanding of the developmental trajectory of the ANS and symbolic number processing skills, thereby getting insight into whether a deficit in the ANS precedes or is preceded by impaired symbolic and exact number processing. Recent work has also suggested that humans are endowed with a shared magnitude system (beyond the number domain) in the brain. We therefore investigated whether children with DD demonstrated a general magnitude deficit, stemming from the proposed magnitude system, rather than a specific one limited to numerical quantity. Fourth graders with DD were compared to age-matched controls and a group of ability-matched second graders, on a range of magnitude processing tasks pertaining to space, time, and number. Children with DD displayed difficulties across all magnitude dimensions compared to age-matched peers and showed impaired ANS acuity compared to the younger, ability-matched control group, while exhibiting intact symbolic number processing. We conclude that (1) children with DD suffer from a general magnitude-processing deficit, (2) a shared magnitude system likely exists, and (3) a symbolic number-processing deficit in DD tends to be preceded by an ANS deficit.

## Introduction

During the past 10 years, researchers have paid increasing attention to the origin and etiology of developmental dyscalculia (DD; prevalence rate = 3.5–7%; Shalev, 2007; Rubinsten and Henik, 2009). Evidence is mounting that DD should be characterized as a weakness or deficit in innate *number sense* or *numerosity coding* (Butterworth, 2005; Wilson and Dehaene, 2007), where both terms refer to a cognitive component responsible for the apprehension and manipulation of numerosities. Number sense and numerosity coding differ in how these manipulations and apprehensions are performed. The number sense hypothesis proposes that the deficit in DD is located in the *approximate number system* (ANS) responsible for representing large and approximate numbers via a logarithmic analog mental number line (Feigenson et al., 2004; de Hevia et al., 2006; Dehaene, 2011). In contrast, the numerosity-coding hypothesis states that DD is caused by a deficit in the processing of smaller and exact sets of numbers (Butterworth, 2010).

This preverbal ability to represent and manipulate quantities may constitute the foundation for the symbolic number system used for learning formal arithmetic (e.g., Dehaene, 2011). As young children develop language and a language-based symbolic number system (i.e., counting words and digits), it is believed there is a mapping of the counting words and visual symbols onto the innate number system (Starkey and Cooper, 1980; Gallistel and Gelman, 1992; Wynn, 1992, 1995; Xu and Spelke, 2000; Feigenson et al., 2004; Gelman and Butterworth, 2005; Piazza, 2010; Dehaene, 2011).

Children with DD have been found to be impaired on non-symbolic number comparison tasks that presumably tap into preverbal number sense (Dehaene, 2011; Mazzocco et al., 2011). Piazza et al. (2010) provided further empirical support for the hypothesis that children with DD demonstrate a deficit in the ANS. They estimated and compared the acuity of the number sense system by calculating Weber fractions (*w*) of performance on non-symbolic magnitude discrimination tasks, which involve selecting the larger of two sets of objects. An important characteristic of the ANS is its general imprecision, which is due to its logarithmic nature. That is, larger numbers are represented closer together than smaller numbers. This makes number discrimination and apprehension of quantities more prone to error as a function of the magnitude and decreased ratio between sets (Dehaene, 1992; Feigenson et al., 2004; de Hevia et al., 2006; Bugden and Ansari, 2011). However, it is believed that with increasing experience with the symbolic system, children learn to compensate for the logarithmic nature of the ANS. Or, the innate ANS is sharpened through the acquisition of the exact symbolic number system (Feigenson et al., 2004; Halberda and Feigenson, 2008; Mundy and Gilmore, 2009; Piazza et al., 2013).

Several neuroimaging studies have also identified the neurocognitive correlates of this innate ability, and converging evidence points to the intraparietal sulcus (IPS) as a focal cortical area of interest (see Kaufmann et al., 2011, for an excellent review of fMRI studies on number processing). The IPS has been implicated in children with DD, where structural and functional differences in gray matter volume and activation patterns have been found (Price et al., 2007; Kaufmann et al., 2011; Ashkenazi et al., 2012).

However, not everyone agrees with this contention regarding the core deficit in DD and the developmental trajectory of number processing (see Rousselle and Noël, 2007; Noël and Rousselle, 2011). Noël and Rousselle (2011) argue against what they call *the simple story* and instead contend that impaired ANS acuity is a result, rather than a cause, of the deficient construction of exact representations of numerical value that builds on symbolic numbers. They provide an overview of multiple studies investigating DD at different ages, and highlight that across the age span, a pattern emerges such that non-symbolic impairment in the ANS is preceded by impairment in symbolic and exact number representations. Impairment in symbolic number processing, which can be observed from ages 6 to 7, together with intact non-symbolic number processing (Rousselle and Noël, 2007; De Smedt and Gilmore, 2011), might subsequently undermine maturation of the ANS representations in DD, leading to impaired non-symbolic approximate number processing around age 10 (Noël and Rousselle, 2011). This developmental perspective builds on their previous *access deficit hypothesis*, which states that children with DD have a core difficulty in relating numerical symbols to their underlying semantic representation (Rousselle and Noël, 2007). Thus, according to this view, and in contrast to the *simple story* outlined above, the developmental trajectory and interaction between the number systems are reversed (see Noël and Rousselle, 2011 and Le Corre and Carey, 2007).

The primary purpose of this study is to enhance our understanding of DD by investigating and contrasting this apparent discrepancy between hypotheses. Although a longitudinal design would be required for a definitive answer, our cross-sectional design may give us insight into this question by using both an aged-matched control group and a younger ability-matched control group. Thus, our goal is to investigate the core deficit as well as the developmental trajectory of number processing skills in children with DD. To answer these questions, we will focus on tasks requiring symbolic number processing, non-symbolic and exact number processing, as well as processing that taps into the ANS.

Another question is whether the ANS is a specialized cognitive domain exclusively dedicated to numerical quantities, or part of a general system for approximating magnitudes across several dimensions, such as space, time and other continua. This contention of a shared system for numbers and time is further fueled by the fact that individuals with DD often complain about their poor perception of time (Cappelletti et al., 2011a).

A secondary purpose of this study is therefore to investigate the nature of this shared magnitude system. Our hypothesis is that children with DD may show impairment on tasks tapping into the ANS and on other tasks pertaining to other dimensions of analog processing, such as time and space. This hypothesis originates from a suggestion by Feigenson (2007) who reasoned that if the diverse magnitude representations share a common mechanism, then deficits in one dimension should be paralleled by deficits in other magnitude processing abilities as well.

Cantlon et al. (2009) suggest that neural processes underlying approximate magnitude processing could originate from a shared evolutionary heritage. Thus, overlapping and distributed neural networks specialized for a given magnitude dimension might stem from a single magnitude system. This original magnitude system might subsequently have been co-opted by other magnitude dimensions throughout phylogeny. Alternatively, dedicated and isolated magnitude systems may have evolved simultaneously and independently from each other, and obey common processing principles due to efficiency or neural constraints (Cantlon et al., 2009). Recently, researchers have paid attention to the nature of this shared magnitude system. The accumulating body of empirical work suggests that a shared core system is responsible for the representations of magnitude of different dimensions, such as time and space, and can be attributed to areas around the parietal cortex (Walsh, 2003; Bueti and Walsh, 2009). This led to the formation of A Theory of Magnitude (ATOM; Walsh, 2003; Bueti and Walsh, 2009). Recent research has focused on how, and to what extent, these representational systems are shared (e.g., Feigenson, 2007; Cappelletti et al., 2011a,b; Kramer et al., 2011; Agrillo and Piffer, 2012; Fabbri et al., 2012). However, there is disagreement about the degree to which these magnitude dimensions are overlapping or dissociated. Some have argued that there is a single internal accumulator representing both duration and sequentially presented numerosity (Meck and Church, 1983). Some theorists have argued for complete independence of each respective magnitude dimension (e.g., Murphy, 1996, 1997), whereas others have proposed partly shared representational mechanisms as well as dimension-specific processes (e.g., Walsh, 2003; Cantlon et al., 2009). The interaction between different magnitude dimensions has been a prime target for investigation. For example, Cappelletti et al. (2011a) found that numbers and time were dissociated in adults with DD, such that participants showed impaired temporal discrimination performance only when numbers were part of the task stimuli. Mussolin et al. (2011) investigated a group of adults with DD and found intact spatial processing on a spatial bisection task. Consequently, they argued that spatial processing is relatively intact in DD and is dissociated from number processing. Conversely, Fabbri et al. (2012) found a triple interaction between all three dimensions. However, they were concerned that the time-numerical interaction effect was somewhat less consistent than the other effects. Thus, they tentatively attributed it to the stimuli used and called for more research on this topic. Further research is indeed warranted, especially given that Cappelletti et al. (2011a) found that numbers and time are at least partially independent magnitude representations and that adults with DD have intact temporal abilities. Interestingly, in a recent study, Vicario et al. (2012) found discrepant results from Cappelletti et al. (2011a). Children with DD were found to have impaired temporal processing skills despite numbers not being a part of the task stimuli.

In a timely review of empirical findings into the nature of the potentially shared representational magnitude system, Bonato et al. (2012) emphasize the heterogeneity of the methods adopted by researchers. They highlight that varying temporal processing intervals have been used and that most studies have been conducted using sub-second intervals. Thus, one alternative interpretation of the results from Cappelletti et al. (2011a) is that temporal ability on the sub-second timescale is dissociated from numbers to some degree and is intact in children with DD. What was not addressed is whether temporal abilities on the supra-second timescale are implicated in DD or part of the shared magnitude system. Although temporal processing may indeed be shared in a distributed neural network, supra-second time processing may be more overlapping in a core magnitude system than sub-second time processing. Emerging neuroscientific data are shedding light on the cortical substrates pertaining to different temporal processing systems, and the processing and representation of time seems to be separate for different time scales (see Buhusi and Meck, 2005, for a detailed overview). These are often divided into three different temporal periods. For example, circadian timing is the representation of the 24-h light-dark cycle that regulates sleep, metabolism, and appetite, and is mainly regulated by hormonal activity in the suprachiasmatic nucleus of the hypothalamus. There seem to be two distinct neural timing systems of smaller time scales. The first automatic timing system is for shorter intervals up to approximately 1000 ms, and recruits the motor systems of the brain (Wiener et al., 2010), such as the supplementary motor area (SMA), basal ganglia and cerebellum (Bonato et al., 2012). The second cognitively controlled timing system is for supra-second intervals and is mainly connected to the prefrontal and parietal cortical areas (Lewis and Miall, 2003; Bonato et al., 2012). The right inferior parietal cortex (rIPC) has been found to be important in time perception, especially concerning the representation and timing of activity within the interval timing scale of space (Buhusi and Meck, 2005; Wittmann, 2009). Indeed, lesions in the PPC may result in deficits in both spatial and temporal tasks (Walsh, 2003). Thus, one alternative interpretation of the findings of Cappelletti et al. (2011a), with respect to sub-second temporal processing in adults with DD, is that they did not tap into the temporal interval implicated in the shared magnitude system. This is further corroborated by findings from Kramer et al. (2011) who determined that time estimation ability within a higher timing interval (100–3000 ms) than the one used by Cappelletti et al. (2011a,b) predicted self-reported mathematical intelligence. Thus, further inquiry is needed into the nature of the shared magnitude system.

Our objective was to enhance our understanding of the etiology and developmental trajectory of number processing in DD. Number processing deficits and domain-general cognitive abilities have already received a great deal of attention (e.g., Feigenson et al., 2004; Butterworth, 2005; Wilson and Dehaene, 2007; Mazzocco et al., 2011). We aim to expand on current knowledge and focus on lesser-known dimensional processing in DD and the proposed shared magnitude system. By comparing a sample of fourth graders with DD with an aged-matched control group of typical achievers (TA4), as well as an ability-matched control group of typical achievers in the 2nd grade (TA2), the severity of impairments on different tasks and the relative development of the number processing systems can be determined. To this end, we developed a novel time discrimination task that targeted the interval timing scale, hypothetically eliciting activation patterns in and around the IPS, which is also implicated in number processing (Kaufmann et al., 2011) and spatial processing, such as mental rotation (Kucian et al., 2007). Number processing in DD was investigated using a non-symbolic number discrimination task and a dot-counting task. Spatial processing was investigated using a traditional mental rotation task together with a paper-folding task.

If DD can be attributed to a primary deficit in the ANS, children with DD should exhibit impaired performance on the non-symbolic number discrimination task compared to TA4, after controlling for domain-general cognitive abilities. We also expect them to exhibit impaired non-symbolic number discrimination performance compared to TA2, but not necessarily on symbolic number processing. Through training, exposure, or other compensatory mechanisms, the DD group may display symbolic number processing on par with TA2.

If the deficit in the ANS is a result of a compromised exact symbolic system, in line with Noël and Rousselle (2011) and their alternative developmental account, we expect impaired symbolic and non-symbolic number processing compared to TA4. This is because the symbolic and exact number system should be the primary dysfunction, which subsequently prevents maturation of the ANS representations in children with DD. However, compared to TA2, we also expect that the DD group will show impaired symbolic number processing abilities but no difference with respect to ANS acuity as measured by the non-symbolic discrimination task. This is because poor symbolic processing should be the primary cause of DD with ANS deficit being a later consequence.

We also hypothesized that the DD group, in accordance with the ATOM account, would show impaired cognitive processing in all three dimensions (space, time, and number) compared to TA4. TA2 was also used to investigate the severity of impairments on these tasks and the relative development of symbolic and non-symbolic systems. In turn, these findings may indicate whether DD is caused by, or results in, a deficit in the ANS. An amalgam of tests tapping into various domain-general cognitive abilities, such as non-verbal intelligence and visuospatial working-memory capacity, was also administered to control for these effects.

## Materials and methods

### Participants

Participants were 82 Swedish schoolchildren (28 boys and 54 girls) enrolled in the fourth grade (*N* = 51, mean age = 10.54, *SD* = 0.40) and second grade (*N* = 31, mean age 8.79, *SD* = 0.29) for whom Swedish was the primary language. They were recruited from 14 different schools in an urban Swedish school district, primarily consisting of middle-class families. Parents gave informed consent for their children to participate. All participants had normal or corrected-to-normal vision and normal color vision. We excluded children with a history of neurologically based impairments, such as ADHD or other known learning disabilities (e.g., dyslexia).

The participants were divided into three groups: children with DD enrolled in their 4th year, a control group consisting of children with mathematical ability typical of 4th year students in the Swedish educational system (TA4), and an ability-matched control group of typical second graders (TA2). Group allocation followed the approach used by Andersson and Östergren (2012). To be included in the DD group, a child had to receive special education in mathematics while showing no need for such special education or instruction in any other subject. In addition, the child had to perform at or below 1.5 *SD* from the mean on the mathematics screening test battery, comprising two separate subtests, compared to the age-matched controls of typically achieving children. This cutoff criterion can be considered quite conservative, given that many studies employ a more liberal cutoff, such as 1 *SD* or below the 15th percentile (15th percentile: Rousselle and Noël, 2007; 25th percentile: Jordan et al., 2003; 35th percentile: Geary et al., 2000). Another justification for using this cutoff criterion is that it would roughly correspond to incidence estimates of DD in the general population (i.e., 5–10%). Although children with learning disabilities such as dyslexia were excluded, a screening of reading ability was used to ensure that children did not have undiscovered impairments, given the high comorbidity between math and reading disabilities. Thus, to be included in the DD group, the children also had to perform within 1.5 *SD* of the mean on a reading test (Malmquist, 1977). To be classified as typical achievers and included in TA4, children had to perform within 1 *SD* of the mean for both math and reading ability. Children in the TA4 group were recruited from the same classrooms as children in the DD group, to minimize effects of instruction or environmental influences that may affect learning and ability. Participants in the TA2 group were selected from the same schools as their older peers, based on teacher recommendations of students who performed within the average range across school subjects. Ultimately, 19 children (6 male, 13 female; mean age = 10.52 years; *SD* = 0.45) were assigned to the DD group, 32 children (11 male, 21 female; mean age = 10.54 years, *SD* = 0.38) were assigned to the TA4 group, and 31 children (11 male, 20 female; mean age = 8.79 years; *SD* = 0.29) were assigned to the TA2 group. An overview of these descriptive data can be found in Table 1.

**Table 1:** **Descriptive participant information by group**.

	**DD**		**TA4**		**TA2**	
	***M***	***SD***	***M***	***SD***	***M***	***SD***
N (number of boys)	19 (6)		32 (11)		31 (11)	
Mean age (in years)	10.52	0.46	10.54	0.38	8.79	0.29
Mathematics score	9.95	3.95	35.41	8.68	13.03	8.74
Reading score^*^	8.68 (11)	1.73	14.94 (54)	4.19	7.06 (–)^a^	3.80
RPM^*^	18.42 (29)	6.17	25.69 (60)	3.30	21.55 (60)	5.32

DD, Children with mathematical difficulties in 4th grade; TA4, Age-matched control group enrolled in 4th grade; TA2, Ability-matched control group enrolled in 2nd grade.

* Raw scores with mean percentile in brackets.

a Normative data unavailable.

### Screening tests

#### Mathematical ability

This was determined using (1) a paper-and-pencil test (with two subtests) involving arithmetic calculations, and (2) a computer-based test that required retrieval of arithmetic facts.

The first subtest of the paper-and-pencil test consisted of eight addition and eight subtraction problems (written in Arabic numerals) that became progressively more difficult to solve (e.g., 57 + 42; 4203 + 825). Children had to solve as many problems as possible within the allotted time (10 min) using only the paper and pencil at their disposal. The second subtest consisted of 12 equations to be solved. These equations were written as arithmetic problems in Arabic notation, which involved subtraction, addition, and multiplication, and where one of the operands was missing (e.g., 61 + ___ = 73). Children had to solve as many of these equations as possible within 7 min. Participants received no feedback about the correctness of their answers. In total, the paper-and-pencil test contained 28 items, and each problem that was solved correctly yielded one point. Consequently, the maximum possible score on this test was 28.

The computer-based arithmetic fact retrieval task consisted of 36 arithmetic problems presented on a computer screen individually. Children had to solve these problems as quickly as possible by retrieving the fact from memory and providing the answer verbally to the experimenter. Response times (in milliseconds) were recorded by the experimenter using the SuperLab environment. Children were not provided feedback about the accuracy of their answers. The arithmetic problems in this test were easy (e.g., 5 + 3); a majority of the participants could provide the correct answers within 3 s. Thus, this task measured the direct retrieval of arithmetic facts from long-term memory rather than calculations. The number of problems answered correctly within 3 s was used as a dependent measure (cf. Russell and Ginsburg, 1984), with a maximum score of 24. Mathematical ability was measured using a single composite score based on the computerized test and the paper-and-pencil test, which resulted in a maximum score of 52.

#### Reading ability

This test consisted of one short story to be read by the participant. The narrative took the form of a fairy-tale, and scattered throughout the text were single missing words replaced by a blank space and a bracket containing four words. When children arrived at a missing word, they had to select which of the words made the most sense in terms of the sentence and the story, and underlined their answer. This reading test contained 20 items (i.e., missing words) scattered evenly throughout the story. The number of correctly selected items within 4 min was the measure of reading comprehension and speed.

#### Non-verbal intelligence

Raven’s Standard Progressive Matrices (Raven, 1976) was administered to groups of four children at a time. Due to the overall mental demands of this study on the children, involving a multitude of tasks over a significant amount of time, only Sets B, C, and D were used to measure non-verbal IQ. The raw scores were converted to percentiles to show how the children performed in relation to standardized scores. The raw scores were used as a covariate in subsequent analyses.

### Domain-general cognitive abilities

#### Color naming

The rapid automatized naming task (RAN) was used to assess participants’ speed of access to semantic information in long-term memory (Temple and Sherwood, 2002). Two sheets of paper were used, and the string “XXX” in Arial 22-point font was printed in different colors—red, green, blue, black, and yellow—and in two separate columns for a total of 30 XXX-strings. Participants had to name the color in which the strings were printed as fast as possible without making any errors. The experimenter continually checked for any errors throughout the trial, registered whenever they occurred, and used a stopwatch to measure the total response time, which was also used as a dependent variable.

#### Visuospatial working-memory

The computerized visuospatial working-memory task consisted of a matrix of 2.5 × 2.5 cm squares. Viewing distance from the screen was 50 cm. The number of squares in each matrix varied and increased, making each subsequent trial more difficult. Initially, the matrix consisted of 3 × 3 squares, where one square contained two black dots. Participants were asked to estimate whether these dots were of equal size, and press the “^*^” key if they were equal or the “A” key if they were not. They had 3 s to respond, after which two additional dots appeared in another square while the former two dots were still visible. Once again, they had to decide whether these two dots were of equal size and respond accordingly within 3 s. In addition to making these judgments, they had to remember in which squares the dots had been presented. The matrix disappeared after a given sequence of dots had been presented, and participants had to mark answers in the corresponding squares on a piece of paper containing an identical matrix. The initial matrix of 3 × 3 squares and two squares with black dots had a trial of span size 2. The subsequent matrix had 3 × 4 squares, and black dots appeared in three squares, giving a span size of 3. In this way, the task became progressively more difficult, with the sixth and final level including a span size of 7 items. Thus, this task included span sizes between 2 and 7 items. For each span size, there were two matrices presented to the participants. All trials and span levels up to span 5 were presented to participants, regardless of performance on any given difficulty level, and the total number of correctly recalled locations of dots was used as the dependent measure. If a participant successfully recalled at least one trial on span 5, the participant moved on to the two trials at span level 6. If the participant correctly responded to at least one trial at this level, the final level was presented to the participant. Participants did not receive feedback whether they were right or wrong, and had to respond correctly to the question (i.e., the size decision) to receive points for the trials. The maximum score on this task was 54 and was used as a dependent variable.

#### Listening span

This task measured the participants’ verbal working-memory capacity. The participant was orally presented with three-word sentences, where the initial task was to make a judgment of whether the sentence made semantic and syntactic sense. Thus, the participant was to respond “yes” if the sentence made sense (e.g., “The rabbit was fast,” which in Swedish would be “Kaninen var snabb,”) or “no” if the presented sentence was absurd (e.g., “The frog played the piano,” which in Swedish would be “Grodan spelade piano”). Participants were instructed to try to remember the first word in each sentence regardless if the sentence was absurd or not. After they orally answered “yes” or “no,” the next sentence was presented. The first span size level was 2, meaning that participants were read two sentences, after which the participant had to recall, in correct serial order, the target words. The span size ranged from 2 to 5, with two trials for each span size, and all span sizes were presented to the participants. The total number of correctly recalled words was the dependent measure in this task, and as in the visual matrix task, the participant had to identify the sentences correctly. Participants received feedback regarding correctness after each trial. Half of the sentences made sense, and the other half were absurd. Each sentence was read to the participant, word-by-word, at a rate of approximately one word per 0.5 s.

### Measures of number, space, and time

#### Number processing

Various tests of both symbolic and non-symbolic number processing skills were administered.

***Symbolic number comparison.*** Two Arabic single-digit numerals (printed in Arial 40-point font) were simultaneously and horizontally displayed on a computer screen. The center-to-center distance between the two numerals was 10 mm. Participants had to decide which of the two numerals was numerically larger, and respond with either “A,” corresponding to the left numeral, or “^*^” corresponding to the right numeral. Before each trial, a fixation cross was displayed for 1000 ms, after which two digits were presented and remained exposed to the participant until he/she pressed a button. Two numerical distances were used: 1 and 4–5, and each pair was presented twice, resulting in a total of 32 trials. The response times and errors were registered for each trial by the software program, and only the response times for correct responses were recorded and used in the analysis. Responses for which response times were less than 200 ms were discarded and considered guesses or false starts, and response times exceeding 2.5 *SD* of a participant’s mean response time were also excluded (amounting to less than 0.5%). The remaining correct responses within that time interval, which ended up being 94.4% of all trials, were used to calculate a mean response time. No feedback on response accuracy was given.

***Number naming.*** This task was a measure of rapid lexical access to number words. Two sheets of paper were used in this experiment, each sheet containing 1- or 2-digit numerals. In the 1-digit condition, seven rows of the Arabic numerals 1–9 were printed in black ink (Times, 28-point font). Each numeral appeared once in every row, resulting in 63 numerals in all. The setup was similar in the 2-digit condition, with 6 columns and 9 rows of numerals, resulting in 54 items. Participants had to name each numeral as fast as possible without making any errors. A stopwatch was used to measure the total time taken to name all the Arabic numerals. Errors were registered by the experimenter. The aggregated RT from both conditions was used as the dependent measure of digit naming speed.

***Number discrimination***. Individual ANS acuity was measured using a number discrimination task. Participants saw two intermixed arrays of blue and yellow dots for 800 ms, after which they were asked to determine whether there were more yellow or blue dots, and subsequently press a color-coded key on the computer keyboard. Participants sat 50 cm from the screen. Participants received no feedback on response accuracy. This task was almost identical to the one used in Halberda et al. (2008), except for the exposure time of the stimulus presentation. These were extended to suit younger children and based on times used by Halberda and Feigenson (2008). The task contained five practice trials followed by 75 test trials. The arrays contained between 5–16 dots and the ratio of colors varied among four ratio bins (1:2, 3:4, 5:6, and 7:8). Half of the trials contained more blue dots and the other half contained more yellow dots. Moreover, for half of the trials, the total blue and yellow surface area was equal, and the dots varied in size to ensure that numerosity was the critical aspect of the task. However, occupancy (a measure of spatial extent and density that may be used for quantity estimation; Allik and Tuulmets, 1991; Durgin, 1995; Kramer et al., 2011) was not controlled for in the task. By using arrays with progressively more difficult ratios, we were able to determine each participant’s *w* by fitting a psychophysical model to the data as a measure of ANS acuity.

***Subitizing and enumeration.*** In this task, arrays of randomly arranged dots were displayed on a computer screen, and participants had to identify how many dots were present, as quickly as possible without making any errors. Dots varied in quantity between 1 and 8, and each dot had a diameter of 9 mm. Viewing distance was 50 cm. A timer measured the response time, beginning from the stimulus presentation (i.e., the array of dots) to the oral response regarding quantity. After the participant responded, the screen went blank for 1000 ms, and then a subsequent trial ensued without feedback. Throughout the trial, the experimenter registered any errors committed by the participants. Each set of dots ranged from 1 to 8 was presented three times, and there were 24 trials in total. Trials with 1–3 dots were used as an estimate of subitizing speed; trials with 5–8 dots were used to measure enumeration ability. Sets were presented in random order, and the dependent variable was the mean response time.

#### Time

The processing of time was measured using a prospective time discrimination task. The participant was presented with a reference stimulus centered on the screen, in the form of a red ball on a white background. The reference stimulus presentation lasted for 3000 ms, followed by a blank screen for 500 ms, after which a target stimulus (a blue ball) appeared centered on the screen. The task was simply to estimate which of the two stimuli was presented the longest. After the target stimulus disappeared, a response screen followed prompting a response. The reference was always presented for 3000 ms and always before the target, whereas the target stimuli duration ranged from 1500 to 6000 ms, spanning the range of *interval timing* (Buhusi and Meck, 2005). Input was provided using a keyboard, without feedback about correctness. If the participant estimated that the reference stimulus lasted longer, the “a” key (marked with red) was pressed. If the target stimulus was estimated to last longer, the participant pressed the blue-marked “^*^” key. This two-interval discrimination paradigm is similar to the one used in Cappelletti et al. (2011a) but with some notable differences. For example, the stimulus interval duration in the current study ranged between 1500 and 6000 ms, whereas Cappelletti et al. (2011a) applied a sub-second time discrimination paradigm. The ratios between the reference and the targets were such that they corresponded to the Weber fractions consistently found in other magnitude dimensions (e.g., Halberda and Feigenson, 2008) and used in other tests of magnitude discrimination employed in this study and elsewhere. Thus, all trials belonged to four different “bins” corresponding to a specific ratio. The four ratio bins were 1:2, 3:4, 4:5, and 5:6 across 60 test trials, where each participant’s *w* could be determined by fitting a psychophysical model to the estimation ability. The test included four practice trials prior to the recorded trials and the participants were asked not to use any counting strategies, such as sub-vocal counting, during the task.

#### Space

Two separate tests tapping into various aspects of spatial processing were administered: (a) a mental rotation task and (b) a paper-folding task.

***Mental rotation task.*** This was a pencil-and-paper test identical to the one used in Neuburger et al. (2011), which was based on a test originally created by Vandenberg and Kuse (1978). The test involved two sets of stimuli unique to two different subtests: in one subtest, the stimuli consisted of alphabetic letters, and in the second, the stimuli consisted of cube figures adapted from Shepard and Metzler (1971). Each subtest contained 16 items, where the reference was located on the left side accompanied by four comparison stimuli located on the right side adjacent to the target. The comparison stimuli always consisted of two “correct” and two “incorrect” items. The primary task was to identify the two matching items, which prompted a mental rotation, and respond by marking them with a pen. Inverted instances of the target (i.e., visually mirrored) were used as incorrect comparison stimuli. All comparison stimuli were rotated only in the picture-plane and in one of six rotation angles: 45, 90, 135, 225, 270, or 315°. The participants had to mark both correct comparison stimuli to obtain one point for each item, yielding a maximum score for each subtest of 16 and, hence, 32 for the entire test. The time limit was 2 min for the letter condition and 4 min for the figures condition, as the latter condition was more difficult.

***Paper-folding task.*** This spatial visualization task contained 20 items. Each item involved the visual presentation of a square piece of paper being folded a given number of times followed by a hole being punched through the paper, thereby piercing all the layers of the paper. The task was adapted from Ang and Lee (2008), with a subset of items being used from their original work. The task was to imagine how this piece of paper would look when unfolded again. Beneath the folded paper, participants were given five alternatives, one of which was correct. Participants provided answers by marking the letter accompanying each alternative using a pen. Once a question was answered, the participant moved on to the next progressively more difficult question. Difficulty was manipulated by increasing the number of times the paper was folded. The simplest items were folded once and the hardest twice. Participants had 10 min to complete the test.

### Procedure

The study was conducted over two sessions (one group and one individual session) each lasting approximately 120 min (including a mid-session break) within a temporal window of 1 month. All tests were administered in the same order for all study participants and the group session served as a screening phase. Instructions regarding tasks were read aloud from a printed manuscript to ensure that every participant was given identical information. After instruction was provided, at least one practice trial for each test was performed to eliminate any misconceptions and mitigate any concerns about the nature of the upcoming task. Computer-based tasks were run on a laptop, using SuperLab PRO 4.5. During the group session, the following tasks were administered: Raven’s Progressive Matrices, screening test of mathematics, screening test of reading, mental rotation task, and the paper-folding task. All the other tests were performed individually.

### Data analysis

To analyze the data, separate univariate ANOVAs, ANCOVAs, and effect sizes were computed, using group membership as the independent variable in all analyses, to investigate group differences. If Levene’s test of equality of error variances was violated in any of the measures, a Brown-Forsythe correction was applied after which the Games-Howell *post-hoc* test was used to investigate multiple comparisons between groups. If, on the other hand, equal variances could be assumed among groups, a Tukey-Kramer *post-hoc* was used instead. For tests using RT measures, intra-individual trials were examined to remove outliers; *RT*s < 200 ms were removed, as were *RT*s > 2.5 *SD* of the individual within a test or within a block.

## Results

### Preliminary analyses of descriptive screening data

To investigate whether the three groups differed with respect to general intelligence, giving rise to the potential need to control for this variable in subsequent analyses, univariate ANOVAs were conducted to compare the DD group, TA4 and TA2 on the RPM, with number of correctly answered items as the dependent variable. Due to the violation of the equal variance assumption, the Brown-Forsythe correction was used, and a significant effect emerged, *F*_(2, 79)_ = 12.51, *p* < 0.001, partial η^2^ = 0.26. A Games-Howell *post-hoc* test revealed that the TA4 showed superior performance compared to both the DD group and TA2, but no difference between the children in the DD group and TA2 (*p* = 0.175). Separate univariate ANOVAs on the measures of mathematical ability and reading ability also revealed significant group effects after Brown-Forsythe correction, *F*_(2, 79)_ = 103.63, *p* < 0.001, η^2^ = 0.69 and *F*_(2, 79)_ = 48.52, *p* < 0.001, partial η^2^ = 0.52. Games-Howell *post-hoc* tests revealed that TA4 were superior to the DD group and TA2 on both measures (*p*’s < 0.001), whereas the DD group and TA2 did not differ on the measures of mathematical ability (*p* = 0.46) or reading ability (*p* = 0.34).

Although IQ was not an inclusion criteria in this study, the raw scores on RPM were used as a covariate in the ANCOVAs to control for these group differences. Because RPM differed between the groups, for each ANCOVA where those variables were used as covariates, we checked for significant Group × RPM interactions, which would indicate violation of the assumption of homogeneity of regression slopes. An overview of the final groups and their respective characteristics can be found in Table 1.

### Analysis of domain-general cognitive abilities

We investigated whether there were any group differences with respect to RAN scores. An ANCOVA with non-verbal intelligence as a covariate showed that the covariate was not associated with RAN performance (*p* = 0.730), but an effect of group when RT was the dependent variable, *F*_(2, 79)_ = 6.96, *p* = 0.002, partial η^2^ = 0.15. Tukey-Kramer *post-hoc* tests showed that the DD group was slower than TA4 (*p* = 0.002) but not TA2 (*p* = 0.286). TA4 showed better performance than TA2 (*p* = 0.027). Thus, for all subsequent analyses using RAN as a covariate in the ANCOVAs, we verified the assumption of homogeneity of regression slopes.

Visuospatial working-memory performance was investigated using an ANOVA, after discovering a Group × RPM interaction. The ANOVA revealed an effect of group, *F*_(2, 79)_ = 3.72, *p* = 0.029, partial η^2^ = 0.09. Tukey-Kramer *post-hoc* tests showed that the DD group did not differ from either control group (*p*’s > 0.479), but that TA4 exhibited better performance compared to TA2 (*p* = 0.024). There was no difference between the groups with respect to verbal working-memory, as measured by the listening span task (*p* = 0.287), when controlling for non-verbal intelligence. An overview of the results pertaining to each group concerning the domain-general cognitive abilities can be found in Table 2.

**Table 2:** **Overview of domain-general cognitive abilities**.

**Tasks**	**DD**		**TA4**		**TA2**	
	***M***	***SD***	***M***	***SD***	***M***	***SD***
Color naming (RAN)	59.16^*^	14.75	44.88	8.90	53.35	10.82
Visuospatial working-memory	18.74	12.11	20.94	5.84	16.00	3.60
Listening span	17.95	3.82	22.72	6.39	19.55	5.19

DD, Children with mathematical difficulties in 4th grade; TA4, Age-matched control group of typical achievers in 4th grade; TA2, Ability-matched control group of typical achievers in 2nd grade.

* Statistically significant difference (p < 0.05), between DD and TA4.

### Analysis of magnitude processing

Table 3 provides a summary and overview of the results on the tests pertaining to each dimension (space, time, and number). The remaining results will be divided into three brief subsections, each of which is focused on a specific dimension of processing.

**Table 3:** **Overview of magnitude processing**.

**Tasks**	**DD**		**TA4**		**TA2**	
	***M***	***SD***	***M***	***SD***	***M***	***SD***
**NUMBER PROCESSING**						
Symbolic number comparison (ms)	1146	225	959	202	1251	238
Mean error rate (%)	5.9	4.8	4.6	3.8	6.2	5.3
Small distance (ms)	1248	280	1035	241	1347	266
Large distance (ms)	1054	215	891	170	1162	236
Number naming	170.95	49.43	133.59	34.26	182.87	49.43
Number discrimination (*w*)	0.89^**^	0.89	0.26	0.11	0.49	0.32
Number discrimination (hits)	47.95	9.06	56.31	6.89	51.90	0.32
Subitizing (1–3 dots; ms)	1198	156	1181	307	1207	245
Enumeration (5–8 dots; ms)	3560	727	3200	586	3740	616
Mean error rate (%)	3.1	4.4	1.0	3.2	2.2	2.4
**SPACE PROCESSING**						
Mental rotation	9.58^*^	4.19	18.06	5.55	11.32	4.30
Paper folding	8.21^*^	3.55	12.63	2.87	8.90	2.98
**TIME PROCESSING**						
Time discrimination	38.26^*^	6.21	46.28	7.44	40.39	5.62
Weber fraction (*w*)	0.66^*^	0.43	0.28	0.18	0.46	0.30

DD, Children with mathematical difficulties in 4th grade; TA4, Age-matched control group of typical achievers in 4th grade; TA2, Ability-matched control group of typical achievers in 2nd grade.

* Statistically significant difference (p < 0.05) between DD and TA4.

** Statistically significant difference (p < 0.05) between DD and TA2.

#### Number processing abilities

***Number naming.*** To investigate the speed of the decoding and naming of Arabic numerals, an ANCOVA with color naming as covariate was used to determine the influence of general lexical speed. The analysis showed that the covariate, color naming, was significantly related to the speed of naming symbolic numbers, *F*_(1, 78)_ = 30.14, *p* < 0.001, partial η^2^ = 0.24. There was a group effect, *F*_(2, 78)_ = 4.28, *p* = 0.018, partial η^2^ = 0.10. The Tukey-Kramer *post-hoc* test showed that the DD group did not differ from either control group (*p*’s > 0.05) after controlling for general color naming speed. As expected, however, TA4 was faster than TA2 (*p* = 0.048).

***Symbolic number comparison***. Prior to the analysis on this task, outliers regarding RT were removed in the manner described previously, and error rates were calculated for each individual. Three participants had an error rate of more than 20% (two participants in the DD group and one participant in TA2) and were subsequently excluded from further analysis. The mean error rate for all groups can be found in Table 3. All groups had a mean error rate below 6.2% (i.e., <2 errors out of 32 trials).

To test whether there were any differences in performance in general between groups on this symbolic number comparison task, an ANCOVA with color naming as covariate was conducted to control for general speed. Color naming was significantly related to number comparison performance, *F*_(1, 75)_ = 5.99, *p* = 0.017, η^2^ = 0.07. There was also an effect of group, *F*_(2, 75)_ = 8.00, *p* < 0.001, η^2^ = 0.18, and the Tukey-Kramer tests showed that the DD group did not differ from any of the control groups (*p*’s > 0.22) but that TA2 was significantly slower than TA4 (*p* < 0.001).

To investigate if there were any differences with respect to the distance effect between the groups, a 3 × 2 (group × distance) mixed ANOVA was conducted. We found a main effect of distance, *F*_(1, 75)_ = 99.63, *p* < 0.001, η^2^ = 0.57, meaning that participants in general were faster to respond to large numerical distances (1029 ms, *SD* = 237) compared to small numerical distances (1199 ms, *SD* = 293). There was also a main effect of group, *F*_(2, 75)_ = 13.64, *p* < 0.001, η^2^ = 0.26, but no interaction effect, *F*_(2, 75)_ = 0.88, *p* > 0.05, η^2^ = 0.02.

***Number discrimination***. Preliminary analysis of RTs across groups showed a mean RT of 1578 ms (*SD* = 487). The mean raw score was 52.71 (*SD* = 8.48), for the 70.44% of correctly responded trials (75 trials). Group comparisons were performed in two steps using two separate measures. These were (1) correctly responded trials and (2) Weber fraction of ANS acuity.

We conducted an ANCOVA with non-verbal intelligence as a covariate to investigate differences in correctly responded trials. The results showed a significant effect of the covariate, *F*_(1, 78)_ = 8.59, *p* = 0.004, partial η^2^ = 0.10, but no effect of group, *F*_(2, 78)_ = 1.70, *p* = 0.189. Although this measure revealed no difference between the groups, we were interested in the ANS acuity of each group, and we suspected that Weber fractions would be a more sensitive measure of performance on this task.

**Figure 1:**
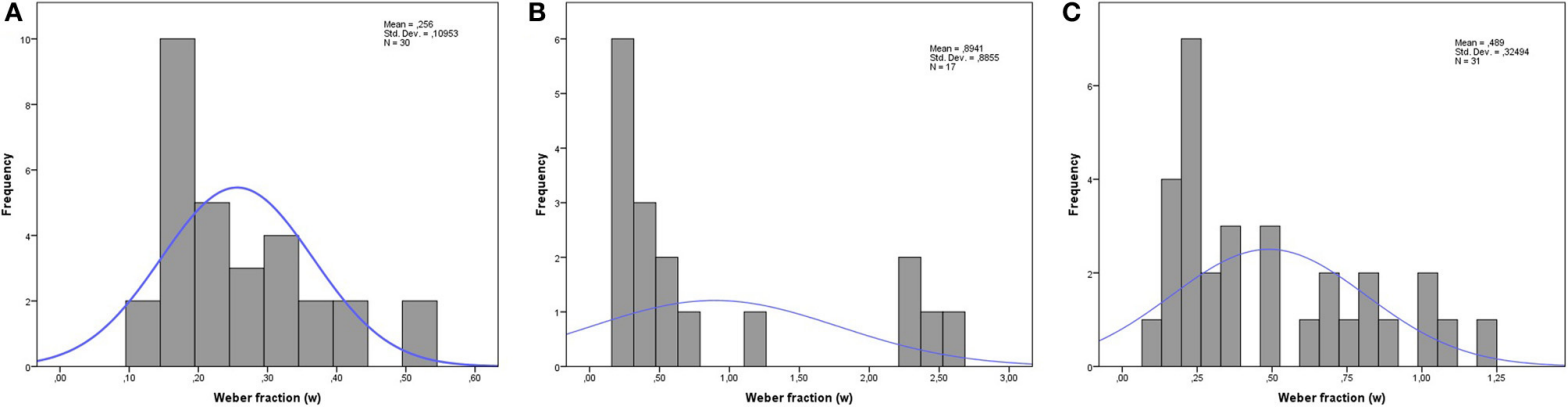
**Histogram of internal Weber fractions on the number discrimination task for (A) TA4, (B) DD, and (C) TA2**.

The acuity of the ANS was first computed and fit using a psychophysics model yielding a Weber fraction (*w*) for each participant, which indicates increased percent correct as a function of the ratios corresponding to the different stimulus bins used in the number discrimination task. This parameter has been used successfully in previous research (e.g., Pica et al., 2004; Halberda and Feigenson, 2008; Mazzocco et al., 2011). The Weber fraction is calculated using the two numerosities as models of two Gaussian random variables, with two means, *n*_1_ and *n*_2_, each with a standard deviation equal to *w* multiplied by the means.By subtracting the smaller Gaussian variable from the larger, a new Gaussian is obtained with a mean of *n*_1_ − n_2_ and a standard deviation of $w\sqrt{n_{1}^{1}+n_{2}^{2}}$. Thus, the accuracy is modeled as 1 minus the error rate, which is defined as the area under the tail of the resulting Gaussian variable: $\frac{1}{2}erfc\left(n_{1}-n_{2}/\sqrt{2w}\sqrt{n_{1}^{2}+n_{2}^{2}}\right)$. Using the Levenberg-Marquardt algorithm for nonlinear least squares fit on the average accuracy for the ratios yielded a *w* for each participant that was an estimate for each participant’s ANS acuity (see Figure 1 for an overview of the results and distribution for each group). Four participants (two in the DD group and two in TA4) received a poor fit to the data (*r*^2^ < 0.2) and as in previous studies (e.g., Starr et al., 2013) these children were excluded from further analysis on this task. We then conducted an ANCOVA with non-verbal intelligence (i.e., raw scores on RPM) as covariate to investigate group differences of ANS acuity. The results showed a significant effect of the covariate, non-verbal intelligence, *F*_(1, 74)_ = 4.04, *p* = 0.048, partial η^2^ = 0.05, as well as an effect of group, *F*_(2, 74)_ = 5.13, *p* = 0.008, partial η^2^ = 0.12. Tukey-Kramer pairwise comparisons revealed that the DD group showed not only significantly noisier ANS representations than TA4 (*p* < 0.01) but also weaker performance compared to TA2 (*p* = 0.042). As can be seen in Figure 1 the distributions of scores are skewed, especially in the DD group. Thus, we also chose to perform a Mann-Whitney *U* comparison of *w* that can handle these unusual cases and skewed distributions. As in the ANCOVA, a comparison between DD group and TA4 revealed a significant difference, *U* = 118, *z* = −3.63, *p* < 0.001, *r* = −0.51. The difference also remained between the DD group and TA2, *U* = 197.5, *z* = −1.94, *p* = 0.052, *r* = −0.27.

***Subitizing and enumeration.*** For all groups, the mean error rate was ≤3%, corresponding to less than one error made throughout the entire task. The mean RT for all participants was calculated and used as a dependent measure of subitizing speed and enumeration speed, after which two separate ANCOVAs using non-verbal IQ and color naming as covariates were performed. The analysis of subitizing speed revealed that non-verbal IQ was associated with subitizing speed, *F*_(1, 77)_ = 5.43, *p* = 0.022, η^2^ = 0.07. In contrast, general speed as measured by the color naming task was not related to subitizing speed (*p* = 0.645) nor was there an effect of group (*p* = 0.569). Analyses of the performance on the enumeration range revealed that color naming speed was related to enumeration speed *F*_(1, 77)_ = 4.16, *p* = 0.045, η^2^ = 0.05, whereas non-verbal IQ was unrelated (*p* = 0.768). There was an effect of group, *F*_(2, 77)_ = 3.52, *p* = 0.035, η^2^ = 0.09. Pairwise comparisons showed that the DD group did not differ from either control group (*p*’s > 0.05) but that TA4 showed faster performance compared to TA2 (*p* = 0.036).

#### Spatial processing

***Mental rotation***. The mental rotation ability of the respective groups was investigated using an ANCOVA with non-verbal intelligence as the covariate and number of correctly answered items as the dependent variable. We found that the covariate was unrelated to mental rotation ability (*p* = 0.256), but there was a significant effect of group, *F*_(2, 78)_ = 14.83, *p* < 0.001, partial η^2^ = 0.28. Tukey-Kramer pairwise comparisons revealed that weaker mental rotation ability in the DD group than TA4 no difference in this ability between the DD group and TA2. Unsurprisingly, the TA4 group performed better than TA2 (*p* < 0.001).

***Paper folding.*** In the same vein, differences in group performance on the paper-folding task were investigated using an ANCOVA with non-verbal intelligence as the covariate and correctly answered items as the dependent variable. In contrast to mental rotation ability, non-verbal intelligence was related to paper folding performance, *F*_(1, 78)_ = 20.57, *p* < 0.001, partial η^2^ = 0.21. There was also a group effect, *F*_(2, 78)_ = 6.13, *p* = 0.003, partial η^2^ = 0.14. Tukey-Kramer *post-hoc* tests showed that the DD group performed on par with TA2 but had significantly poorer performance than TA4 (*p* = 0.042).

#### Time processing

**Figure 2:**
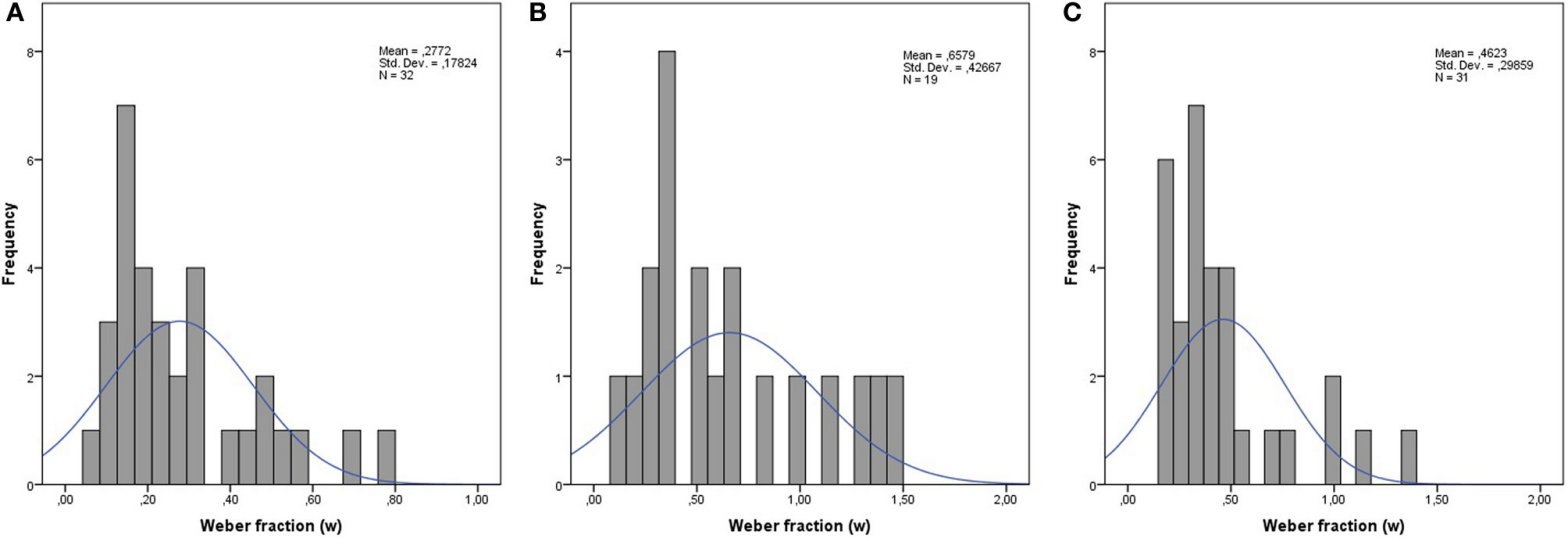
**Histogram of internal Weber fractions on the time discrimination task for (A) TA4, (B) DD and (C) TA2**.

**Figure 3:**
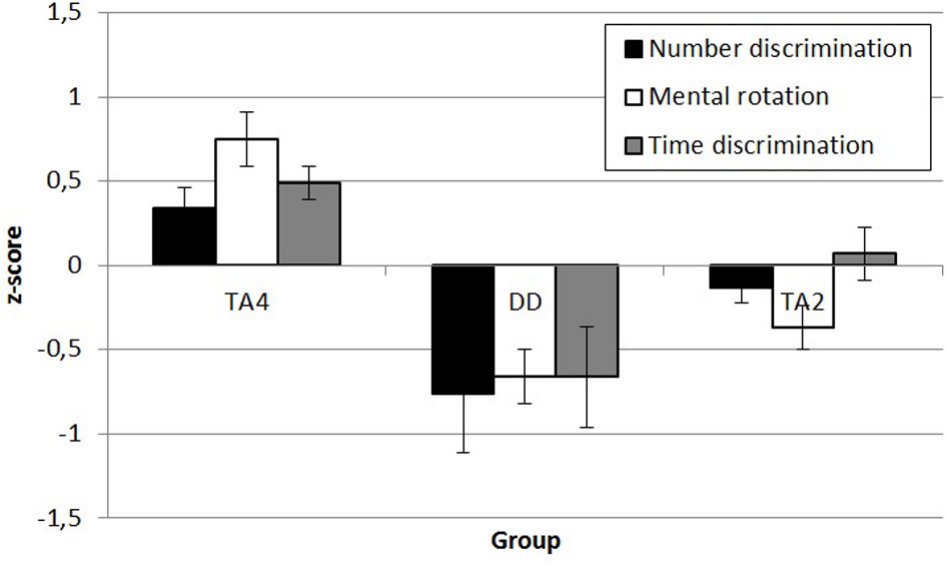
**Overview of z-transformed magnitude processing scores for each group**.

***Time discrimination.*** Our goal was to compute individual *w*’s for each participant and then compare across groups. However, the *w* for temporal magnitudes for adults have not been confirmed in the literature as a valid construct to our knowledge. Therefore, we decided to compare raw scores of the groups on this task as a first step, after which a computation and comparison of *w* ensued. Non-verbal intelligence and visuospatial working-memory capacity were used as covariates in an ANCOVA to investigate the group differences in the raw scores on the time discrimination task. The results showed that neither non-verbal intelligence nor working-memory capacity were related to time discrimination performance (*p*’s > 0.05), but there was an effect of group, *F*_(2, 77)_ = 5.47, *p* = 0.010, partial η^2^ = 0.12. Pairwise comparisons showed no difference between the DD group and TA2, but compared to TA4 there was a significant difference in performance (*p* = 0.009). TA4 also performed better than TA2 (*p* = 0.033). The next step was to calculate the *w* for each individual using the same computational procedure as with the number discrimination task (see Figure 2 for an overview of the results and distribution for each group). Then, we compared the groups by means of ANCOVA with the same covariates as with the raw scores. The ANCOVA showed that the group effect remained when using *w* as the dependent variable, *F*_(2, 77)_ = 4.87, *p* = 0.010, partial η^2^ = 0.11. The pattern of group differences was the same, where the DD group showed poorer performance than TA4 and a small (but not significant) difference from TA2 (*p* = 0.102). Figure 3 provides a visual summary of transformed z-scores across dimensions.

## Discussion

The current study sought to advance our understanding of the etiology and developmental trajectory of number processing in DD, and whether magnitude-processing abilities share representational systems or are subserved by the same mechanism. The first objective was accomplished by comparing a sample of children with DD enrolled in 4th grade to age-matched controls as well as younger ability-matched controls enrolled in 2nd grade. Contrary to Noël and Rousselle (2011), who argue that an ANS deficit is a *result* rather than cause of deficient symbolic number processing, we found support for the hypothesis that DD is due to a deficit in the ANS that is prior to any deficit in symbolic number processing. In line with our hypotheses, we found that children with DD were impaired on all three magnitude-processing abilities. We found that children with DD have a deficit in temporal processing in the supra-second range and inferior spatial visualization, as measured by the paper-folding task and mental rotation ability. These novel findings regarding spatial processing contrast with previous work by Soltész et al. (2007), who found intact mental rotation ability in adolescents with DD. Mathematical problem-solving requires several domain-general cognitive processes. Because several cognitive components are involved in this problem-solving chain, researchers have proposed almost as many possible key components underlying DD. For example, one recurrent finding is that DD in children is a result of a deficit in visuospatial working memory capacity or listening-span tasks, which may function as a bottleneck during mathematical processing (Geary et al., 1999, 2004; Andersson and Lyxell, 2007; D’Amico and Passolunghi, 2009; Andersson, 2010). In the current study, however, we did not seek to investigate these capacities in DD but rather control for their potential influence on tasks relevant to magnitude processing. It is worth mentioning, however, that the children with DD did not show any impairment in visuospatial working-memory capacity or on the listening-span task after controlling for non-verbal intelligence.

### The impaired approximate number system—cause or effect of DD?

One prominent hypothesis concerning the underlying cause of DD in children is that they have a deficit in innate preverbal number sense, which in turn leads to difficulties in developing mathematical competence. A substantial body of empirical work supports this claim (Starkey and Cooper, 1980; Gallistel and Gelman, 1992; Wynn, 1992, 1995; Xu and Spelke, 2000; Gelman and Butterworth, 2005; Landerl et al., 2009; Piazza, 2010; Dehaene, 2011). However, Noël and Rousselle (2011) argue against what they call the *simple story*, contending that impaired ANS acuity is a result of deficient symbolic and exact manipulation of numbers. To investigate this, we administered both a non-symbolic number discrimination task as well as a symbolic number comparison task. We were interested in whether children with DD would show impaired ANS performance compared to age-matched controls, as has been demonstrated in the literature (e.g., Landerl et al., 2009; Piazza, 2010; Mazzocco et al., 2011). Children in the DD group showed a noisier ANS acuity compared to TA4, when the Weber fraction was used as a dependent variable on this task. The resulting Weber fractions were higher than expected (*w* = 0.89 for DD and 0.26 for TA4), and high in relation to those found by Piazza et al. (2010): 0.34 for DD and 0.25 for 10-year old control children. The task used in the current study is different from the one used by Piazza et al. (2010) and the variation in Weber fractions might be due to task difficulty. In the current study, participants saw two intermixed arrays of blue and yellow dots for 800 ms. Discriminating intermixed arrays is likely more difficult than discriminating two separate arrays. Halberda et al. (2008) used a task identical to ours except for the stimulus duration, and reported a Weber fraction of 0.28 in a sample of 14-year old adolescents (range:0.12–0.56). Thus, Weber fractions appear to depend heavily on the way the task is structured. Although the DD group showed impaired ANS acuity compared to TA4, the actual Weber fractions that we report here may not be generalizable to other tasks with other setups. An ongoing debate concerns the different ways in which one can measure ANS acuity. Inglis and Gilmore (2014) investigated four different indices of ANS acuity, where relatedness between measures and their respective test-retest reliability were assessed. The authors raised the concern that Weber fractions consistently showed positive skewness of the distribution that subsequently led to poor test-retest reliability. This finding led to the recommendation that researchers should instead use accuracy as an index of ANS acuity, which showed higher reliability. In our study, we did indeed find a positive skew that urged us to make additional analyses that are not sensitive to skewness. A Mann-Whitney U analysis, which is sensitive to rank rather than actual Weber scores, still indicated poorer performance by the DD group compared to controls. Regarding the validity of different indices of ANS acuity, Inglis and Gilmore (2014) also concluded that both accuracy and Weber fractions are valid indices of ANS acuity, given their strong relatedness (*r* = 0.79). In addition, Inglis and Gilmore (2014) found that children showed a 1-week test-retest reliability of the Weber fraction of *r* = 0.41, whereas the reliability score was *r* = 0.47 for accuracy. Thus, it is debatable whether accuracy really is a substantially more reliable measure than Weber fraction in children. We therefore conclude that our use of Weber fraction as a measure of ANS acuity is justified.

Occupancy was not explicitly controlled for in this task. Occupancy is a measure of spatial extent and density that may be used for quantity estimation (Allik and Tuulmets, 1991; Durgin, 1995; Kramer et al., 2011). Durgin (1995, 2008) argues that numerosity estimation is mainly a perceptual phenomenon, which is governed by density perception. According to this view, the human perceptual system does not detect dots as distinct objects, but rather as parts of a texture by computing summary statistics of dot density. In the current study, quantity discrimination could potentially have been based on the perceptual quantities of blue-dot occupancy and yellow-dot occupancy instead of the cognitive quantities of blue-dot numerosity and yellow-dot numerosity. We cannot distinguish between the two possibilities. However, because our conclusions do not hinge on this matter, we did not investigate it further.

Somewhat surprisingly, the DD group did not show higher RTs nor poorer accuracy on symbolic number comparison when controlling for both non-verbal intelligence and general speed (RAN). However, the result of this one-digit comparison task is in line with previous findings (Ashkenazi et al., 2009). Digit comparison has consistently been tied to DD and general math achievement (e.g., Rousselle and Noël, 2007; Landerl and Kölle, 2009; Andersson and Östergren, 2012). Since the DD group performed worse on measures of ANS acuity, and given the mapping of Arabic numerals and the ANS, these children would logically be impaired on symbolic tasks as well. Perhaps this is due to a compensatory mechanism whereby an affinity for lower numbers due to greater exposure in the school setting has resulted in adequate number processing skills in these lower ranges. Administration of other symbolic comparison tasks, such as two-digit comparison, which could be regarded as more atypical than one-digit comparison tasks, might have shed light on this issue. Other symbolic tasks, such as a two-digit task, might be more sensitive to actual differences between groups and not as easily subject to compensatory processes without targeted practice. In addition to the intact symbolic processing, the DD-group did not show impaired accuracy or slower performance on either the subitizing range or the enumeration range compared to either control group. By comparing the DD-group to an age-matched control group of typical achievers, and in the light of current results on symbolic and non-symbolic number processing, we provide support for the notion that children with DD have an ANS deficit.

By also using a control group of ability-matched second graders, it is possible to obtain some clues as to the severity and developmental trajectory of each of these number-processing skills, and hence obtain valuable information as to whether an ANS deficit precedes, or is preceded by, a dysfunction in the exact representation of symbolic numbers. When comparing the DD group to TA2, we found that they did not differ in symbolic number comparison, nor did they show a larger distance effect. One might posit that exact non-symbolic number representations, as measured by the subitizing and enumeration task, might be impaired, but the DD group did not differ from TA2 on either of these measures.

On the non-symbolic number discrimination task, the DD group showed a noisier ANS than TA2. This suggests that they compensate, perhaps through exposure to culturally derived numerical symbols, and are able to perform adequately on some symbolic tasks, such as the one-digit comparison task in our study. The cognitive profile and pattern of results in this study is not compatible with Noël and Rousselle (2011). According to their hypothesis, we would expect the DD group to show worse performance on symbolic and exact number processing, which would subsequently be responsible for their eroded ANS ability.

Given that children with DD showed impaired magnitude processing on analog magnitude dimensions other than numerical quantity, they likely have a general magnitude-processing deficit, including the ANS, which interferes with accurate symbolic mappings and mathematical competency. Research on preverbal infants and ANS acuity shows that infants who made finer non-symbolic number discriminations at 6 months of age also showed superior and sharper ANS acuity at 9 months of age (Libertus and Brannon, 2010). In addition, ANS acuity at age 3 or 4 years, before taking part in formal mathematics instruction, has been shown to be predictive of standardized math scores at ages 5 or 6 (Mazzocco et al., 2011). Taken together with our current findings, it is likely that ANS representations play a causal role in the acquisition of mathematical ability, which has also been suggested by Feigenson et al. (2013).

Thus, the current results support the simple story, where a deficient ANS in DD leads to noisier number representations. These then affect the foundation for the manipulation of culturally derived Arabic numerals and mathematical processing (Wilson and Dehaene, 2007; Wilson et al., 2009; Piazza, 2010). The answer may also lie somewhere in between these two extremes, in that the ANS acuity is driven by both maturation of brain systems as well as culture and experience (Geary, 2013), in which ANS acuity is sharpened as a result of exposure and manipulation of exact numerical representations. Experience with mathematical material likely sharpens and calibrates the ANS to some extent, given that innumerate cultures have been shown to a have slightly less accurate ANS representations (Pica et al., 2004). In a recent study, Nys et al. (2013) investigated adults with varying educational experience with math, and found that adults who had received formal math education showed superior number processing performance and better approximate number skills. Thus, it is plausible that ANS acuity and mathematical experience have a bidirectional relationship, but that ANS deficits most likely precede symbolic number processing impairments and subsequent mathematical difficulties. A definitive resolution regarding the developmental trajectory of number processing skills in children, such as how ANS acuity develops relative to exact symbolic processing proficiency would require a longitudinal design, in which a sample of children would be assessed on these measures at multiple points throughout ontogeny.

### Magnitude processing as a shared representational system

In addition to the hypothesis of an impaired ability to process numerical quantity, we investigated processing in other dimensions of magnitude, hypothesizing that they would be impaired as well. This hypothesis rests on the assumption that dimensions of magnitude share neural activation patterns centered around the IPS (Bueti and Walsh, 2009). Spatial processing has been found to share activation patterns in the IPS with non-symbolic processing (Kaufmann et al., 2008). Kucian et al. (2007) also found that mental rotation tasks activate cortical substrates in the IPS. Therefore, we hypothesized that the DD group would show impaired mental rotation ability compared to their age-matched peers. They did show poorer performance, with a quite large effect size (partial η^2^ = 0.28), even when controlling for non-verbal intelligence. Glass et al. (2012) found that the right inferior parietal lobule (bordering the IPS) and the left parahippocampal regions were involved during mental paper folding. In addition, mental paper folding is a complex visuospatial ability requiring several sequential mental transformations. Therefore, we hypothesized that the DD group would be impaired on this ability as well. Non-verbal intelligence was significantly related to performance on this task, most likely pertaining to the number of sequential mental operations and transformations that have to be performed. Even when controlling for intelligence, the DD group showed impaired performance relative to their age-matched peers, further corroborating the notion that spatial processing is implicated in DD and likely shares neurocognitive resources or mechanisms with number processing.

With respect to time, we hypothesized that temporal processing within the interval timing scale of space (i.e., supra-second timing) would be implicated in DD and related to the other magnitudes. This hypothesis is based on the fact that neural substrates subserving supra-second processing have been located in prefrontal areas as well as the rIPC (Wittmann, 2009), and that lesions in PPC have been found to result in spatial and temporal processing deficits (Walsh, 2003; Buhusi and Meck, 2005). When comparing the DD group and the TA groups, it was necessary to control for both non-verbal intelligence as well as visuospatial working-memory because it was suspected that supra-second intervals, especially at the higher end of the temporal spectrum, would place a significant load on working-memory resources. Indeed, supra-second temporal processing activates prefrontal areas (Wittmann, 2009), which in turn are cortical loci for spatial working-memory processing together with connecting areas in parietal regions (Rotzer et al., 2009; Rubinsten and Henik, 2009).

As expected, comparisons between all three groups revealed that the DD group showed impaired performance relative to their age-matched peers. To our knowledge, individual Weber fractions as an index of temporal acuity have not been assessed in school-aged children or adults. Infants, however, have been subject to investigation. For instance, van Marle and Wynn (2009) found that 6-month old infants could make temporal discriminations of 1:2 ratios, and Brannon et al. (2007) showed that 10-month old infants could succeed in discriminating duration ratios of 2:3. We calculated Weber fractions for school children enrolled in 4th and 2nd grade (aged 8–10), and found mean temporal acuities of 0.28 for 10-year-olds and 0.46 for 8-year-olds. Interestingly, children with DD had a mean temporal acuity of 0.66, which was significantly higher than that of TA4, and higher (but not significantly) than that of TA2 (*p* = 0.102). This is the first evidence that time processing in the supra-second range is implicated in children with DD regardless of whether numbers are part of the task stimuli, contrary to previous arguments (Cappelletti et al., 2011a,b). In addition, supra-second temporal processing may share a distributed neural network with other magnitudes.

Longer time intervals, exceeding 1.2 s, may allow participants to use counting strategies such as sub-vocal counting, rhythmic breathing, or heart rate (Grondin, 2010). We tried to minimize the use of counting strategies by instructing children not to count in their heads and instead trying to sense which stimulus appeared longer. Even if some children disobeyed, it is difficult to reliably count and discriminate between durations used in the current study, except for the easiest ratio (3000 vs. 6000 ms) where all children performed at ceiling. To investigate the influence of longer time intervals, we re-analyzed how the groups performed after excluding trials exceeding a 1200-ms difference between the reference and the target. The same pattern of results remained. Thus, we conclude that counting strategies did not confound the results.

Studies investigating DD and time processing in the supra-second range are scarce, but Vicario et al. (2012) found that supra-second timing was spared in children with DD, whereas sub-second timing was affected. This discrepant finding may be attributed to the timing interval used. Vicario et al. (2012) used reference stimuli and target stimuli between 1280 and 1520 ms, which is a narrower interval than in our study (1500–6000 ms), as well as the study by Kramer et al. (2011; 100–3000 ms), where they found a relationship between mathematical intelligence and supra-second processing skills. Vicario et al. (2012) tested both sub-second and supra-second intervals using brief temporal gaps between reference and task stimuli, and concluded that temporal deficits in the sub-second range in DD might stem from impaired sensory processes (Vicario et al., 2012). The authors also conclude from their time reproduction task that temporal processes relying on motor systems might be spared in DD while temporal processes in the *perceptual* domain are not (Vicario et al., 2012). We extend findings regarding the temporal processing profile of children with DD. Our results show that temporal processing in the *cognitive* domain (Lewis and Miall, 2003; Bonato et al., 2012), which relies on neurocognitive activity around the IPS and rIPC, and has consistently displayed reduced cortical activity as well as structural anomalies in DD (Kaufmann et al., 2011), is impaired.

Further research is warranted to pinpoint the relationship between the different magnitude dimensions and different temporal intervals. Additionally, our results are in accordance with the inferences made by Cappelletti et al. (2011a) regarding the relative independence of time processing abilities from working-memory deficits.

In sum, the DD group clearly shows impaired magnitude processing across dimensions. As Feigenson (2007) reasoned, if the diverse magnitude representations share a common mechanism, deficits in one dimension should be paralleled by deficits in other magnitude processing abilities as well. One tentative conclusion from the current study is that because all the dimensions are implicated in DD, Feigenson’s (2007) hypothesis is supported, and they share representational mechanisms. The current study did not address any interactions between dimensions, however, which limits the inferential power with respect to the degree of overlap and directionality of associations between dimensions. Thus, we remain hesitant in making any substantial claims about the *structure* of a shared magnitude system, yet we argue that the current results support the existence of one (Walsh, 2003; Bueti and Walsh, 2009; Fabbri et al., 2012), and that it is implicated in DD. It is highly unlikely, given the current results, that magnitude processing is performed by independent domain-specific processes, as suggested previously (e.g., Murphy, 1996, 1997). Thus, the etiology of DD can be traced to a deficit in a more general magnitude processing system than merely to a difficulty with the apprehension and manipulation of approximate numerosities in the ANS. The investigation of causal factors and neurocognitive underpinnings of DD has made important headway, and the current study contributes to the unveiling of the mechanisms underlying DD. Our study points to cognitive processes pertaining to magnitude, which in turn may sub-serve mathematical competency and numeracy. Based on the theoretical implications of this research, we may be able to predict atypical development and identify children at risk of developing DD. Our results also might be leveraged to improve math education and, ultimately, the quality of life for individuals with DD.

## Conclusions

Our study expands on prior knowledge regarding the etiology of DD. While a deficit in the innate and preverbal ANS previously had been suggested to play a role, our results support the notion that impaired ANS acuity in children with DD is the cause, rather than the effect, of impaired exact symbolic number processing. Longitudinal data are needed to confirm these cross-sectional findings. Our findings favor the view that impaired ANS acuity hampers the acquisition and mapping of number symbols, which, in turn, leads to challenges in attaining mathematical competence. The results also indicate that children with DD suffer from a general magnitude-processing deficit that goes beyond the number domain alone. Children with DD showed impaired cognitive processing ability across three dimensions of processing (space, time, and number), and novel findings included impaired mental rotation ability and temporal processing in the supra-second range. The findings that children with DD have a general magnitude deficit also support the notion of a shared magnitude system for representing analog magnitudes across multiple dimensions, which is in accordance with ATOM (Walsh, 2003).

### Conflict of interest statement

The authors declare that the research was conducted in the absence of any commercial or financial relationships that could be construed as a potential conflict of interest.
